# Supplementary material for: Are carbon emissions trading and green financial instruments synergistic? -Comprehensive quantitative research based on content analysis
Source: PLoS One. 2024 Mar 7;19(3):e0298601. doi: 10.1371/journal.pone.0298601 (PMC10919601; doi:10.1371/journal.pone.0298601)
Supplement: S1 Data — (ZIP) [file pone.0298601.s001.zip › Data/地级市Z_F工作报告.zip!/║■▒▒/╥╦▓2╩╨/└┤╫╘╣1⁄2╓┌║┼í╛┬φ┐╦╩2╛▌═°í┐_2016.docx]

各位代表：

　　现在，我代表市人民政府向大会报告工作，请连同《宜昌市国民经济和社会发展第十三个五年规划纲要（草案）》一并审议，并请市政协委员和列席人员提出意见。

　　一、“十二五”时期和2015年工作回顾

　　过去五年，是宜昌市发展进程中极不平凡的五年。面对复杂严峻的宏观环境和自身阶段性特殊困难，在省委、省政府和市委的正确领导下，全市上下深入贯彻落实党的十八大和十八届三中、四中、五中全会精神，牢牢把握新常态下发展主动权，稳神定气，奋进作为，加快推进现代化特大城市建设，圆满完成了“十二五”规划确定的主要目标任务。

　　过去五年，是综合实力大跃升的五年。生产总值连跨两个千亿级台阶，2015年达到3384.8亿元，是2010年的2.2倍，占全省的比重提高1.7个百分点。地方公共财政预算收入连跨三个百亿级台阶，达到339.1亿元，是2010年的4.8倍。全社会固定资产投资连跨三个千亿级台阶，达到3085亿元，是2010年的3.2倍；五年累计完成投资10573亿元，是“十一五”时期的3.6倍。社会消费品零售总额达到1089.5亿元，是2010年的2倍。主要经济指标增幅高于全国、全省平均水平。综合实力跃居全国百强城市第57位，前进16位；跃居中部地区同等城市第2位，前进1位；稳居长江沿线同等城市第4位。宜都晋级全国县域经济百强。宜都、夷陵、枝江、当阳稳居全省县域经济分类考核前列。

　　过去五年，是转型发展大迈步的五年。三次产业结构调整为11：59：30。农业六大特色产业占农业总产值比重达到83%，比2010年提高4.5个百分点。加工产值过百亿元的园区达到4个，销售收入过百亿元的农业产业化龙头企业达到2家。精细化工、食品生物医药、先进装备制造、现代物流四大产业产值分别突破1700亿元、1800亿元、1100亿元和1000亿元，新材料和文化旅游业产值分别达到700亿元和800亿元。规模工业企业达到1491家，产值过亿元企业达到837家，分别比2010年增加684家和568家；产值过百亿元企业9家，比2010年增加6家；3家企业进入中国企业500强，4家企业进入中国民营企业500强。年接待游客4700万人次，实现旅游总收入450亿元，分别是2010年的3.05倍和4.33倍。新增国家5A级景区3家，总量居全国同等城市前列。被评为中国“十佳最具人气旅游城市”、“十佳宜居宜业宜游旅游城市”。新增金融机构31家，存贷款余额分别达到2822亿元、2133亿元，分别是2010年的1.83倍和2.05倍。新增上市企业3家、“新三板”挂牌企业9家。建成各类科技创新平台272家；国家高新技术企业达到273家，比2010年增加218家；高新技术产业增加值达到550亿元，占生产总值的比重达到16%，比2010年提高7个百分点。引进或入选国家“千人计划”专家5人、省“百人计划”专家9人，成立院士专家工作站33家。新增中国驰名商标35件、国家地理标志产品2个、长江质量奖及提名奖5个。成为“中国产学研合作创新示范基地”、“国家创新型试点城市”、“国家知识产权示范城市”、“全国质量魅力城市”，获批创建“全国质量强市示范城市”。全面超额完成省政府下达的节能减排任务。单位生产总值地耗、用水量分别下降30%和33%，省级节约集约模范市（县）实现全覆盖。

　　过去五年，是城乡面貌大变化的五年。中心城区建成区拓展到175平方公里，常住人口增加到175万人。宜昌新区累计完成投资1003亿元，东山大道BRT、峡州大道、点军大道等一批重点基础设施项目，市规划展览馆等一批大型公建项目相继建成，至喜长江大桥、市博物馆新馆、市奥体中心等一批重点项目加快建设，220平方公里新区骨架基本形成，“三年出形象”目标如期实现。宜昌荣膺“世界可持续交通奖”。实施城市整理和绿化美化行动，完成建筑综合整治项目550个、165万平方米，新增绿色建筑222万平方米；城区人均公园绿地面积达到14.29平方米。运河公园整治、东山公园改造、磨基山公园一期等项目相继建成，“六大水系、八大公园”生态景观雏形初显。蝉联六届全省城乡规划建设管理“楚天杯”。五峰县城避险迁建基本完成。14个镇跻身全国、全省重点镇行列。全市常住人口城镇化率达到56.7%，比2010年提高7个百分点。宜巴、保宜高速公路和汉宜城际铁路建成通车，宜张高速公路当枝段和宜五段、宜岳高速公路基本建成。新增高速公路334公里、一级公路408公里、铁路36公里。基本实现县县通高速、国道，乡乡通省道，村村通客车，各县市区全面进入宜昌“一小时经济圈”。三峡综合交通运输体系进入国家战略。三峡现代物流中心基础设施建设项目获2亿美元世界银行贷款支持。三峡枢纽港年吞吐量达到7776万吨；三峡机场年旅客吞吐量达到124万人次，跻身全国机场60强，开通直飞港台和韩国航班。城乡统筹工作走在全省前列。“三万”活动成效明显。建设高标准基本农田166万亩。全市农田灌溉水有效利用系数达到0.52。新增变电容量457万千伏安、35千伏以上线路1031公里。在全省率先实现农村光纤“村村通”。建设美丽乡村示范点8个、宜居村庄100个、特色村庄82个。省级新农村建设示范村、镇达到100个。全市生活垃圾无害化处理率达到96.3%，入选全国首批生活垃圾分类示范城市，餐厨废弃物处理项目建成并试运行。全市森林覆盖率超过65.7%，获得“国家森林城市”称号。

　　过去五年，是改革开放大突破的五年。坚持解放思想，加强顶层设计，连续四年召开大型座谈会，进一步完善现代化特大城市建设的目标体系、基本内涵、发展理念、体制机制和改革部署。把学习对接上海自贸区作为全面深化改革的突破口，加快推进行政审批、简政放权、市场准入、投资制度、社会信用、行业监管等重点领域改革。在全省率先实行“先照后证、三证合一、网上注册、简易注销”，推动企业登记注册全程便利化，市场主体达到50.9万家，是2010年的3.9倍。编制市级“权责清单”，优化行政审批流程，市级投资审批提速80%。食品药品、卫生计生、文化广电等跨领域，宜昌高新区、黄柏河等跨区域的综合执法体制逐步形成，成为全国综合执法体制改革试点城市。组建宜昌新区推进办公室、三峡旅游新区管委会、三峡枢纽港管委会，完善宜昌高新区管理体制，“四大平台”高效运行。调整完善城区财政体制，顺利完成城区建设管理体制、教育管理体制改革。预算管理、政府债务管理等系列财政改革深入推进。改革重组市级国有投融资公司，组建四大控股集团。人福医药与三峡制药、花林建材集团与楚园春酒业等一批企业实现股权重组。深化投融资体制改革，香溪长江大桥、市妇幼医院等一批社会项目成功引入政府与社会资本合作（PPP）模式。梯级土地开发成功破题。农村土地制度、产权制度改革顺利推进，农民专业合作社达到5123家，登记注册家庭农场2021家。文化体制、医药卫生体制、财税体制、政府机构改革、事业单位分类改革等各项改革成效明显。承担新兴产业集聚发展、智慧城市、信息惠民、电子商务等20多项国家试点示范。宜昌市被纳入长江中游城市群规划范围。三峡城市群上升为省级战略。湖北自贸区宜昌片区方案加快申报，三峡保税物流中心获批设立。外贸出口达到26.9亿美元，是2010年的2.1倍；利用外资3.55亿美元，是2010年的1.7倍。五年实际到位招商资金4663亿元，南玻光电玻璃、人福出口药品基地、三宁己内酰胺等一批重大项目相继落户，与三峡集团、武钢集团、中建集团、葛洲坝集团、省交投集团等中省企业战略合作深入推进。安琪集团等40多家企业赴境外投资。三峡库区对口支援工作成效明显，争取对口支援资金477.5亿元。争取三峡后续工作规划国家补助资金42.6亿元。

　　过去五年，是民生福祉大改善的五年。精准扶贫、城乡居民收入倍增、全民创业、社会治理创新、国家食品安全城市创建、城市绿化美化等“六大民生工程”全面实施。城乡居民人均可支配收入分别达到27275元、12990元，分别是2010年的1.75倍、1.89倍。累计减少农村贫困人口28.1万人。五年新增城镇就业56万人，转移农村富余劳动力就业48万人，城镇登记失业率始终控制在3.1%以内。五项保险参保400万人次，基本养老保险、基本医疗保险和新农合参保率分别达到90%、96%和99.8%。企业退休人员基本养老金连续五年上调，城乡低保标准先后五次上调，新农合补助标准、门诊和住院费用报销比例大幅提高。城镇职工基本医疗保险实现市级统筹。城市社区养老服务中心实现全覆盖，农村老年人生活互助照料中心覆盖率超过55%。连续两年进入中国城市“慈善百强”。实施保障性安居工程23.7万套，其中棚户区改造11.8万套；完成农村危房改造7万余户。新解决115.23万人饮水安全问题。连续两年被评为“中国十佳食品安全城市”。整合检验检测资源，组建三峡食品检验检测中心。“一本三化”社会治理创新经验全国推广，在全国率先实现城乡网格化管理全覆盖。

　　以社会主义核心价值观引领精神文明建设，在全省率先获得“全国文明城市”称号，并成功蝉联。文化事业蓬勃发展，入选第三批创建国家公共文化服务体系示范区。5个县市区被命名为“全国文化先进县”。中国长江三峡国际旅游节、屈原故里端午文化节、宜昌长江钢琴音乐节成为全国具有重要影响的节庆活动。200多件文艺作品获省级以上奖励，歌曲《巴土恋歌》《江河恋》获全国“五个一工程”奖。宜昌市被命名为“中国诗歌之城”、“中国钢琴之城”，举办了首届中国屈原诗歌大奖赛。全民阅读指数连续三年居全省同等城市之首。广播电视“户户通、村村响”工程全面推进。群众健身运动蓬勃开展，竞技体育水平不断提高，涌现了一批奥运、亚运金牌选手。坚持立德树人，“宜学之城”建设有序推进。第一期学前教育三年行动计划全面完成。义务教育均衡发展示范县创建实现全覆盖。高中阶段教育全面普及。三峡大学、三峡职院等高校办学水平不断提升。市特校成为中国残疾人艺术团唯一附属学校。在全省率先完成职教资源整合，组建三峡中专、三峡技师学院和三峡职教集团，职教园一期工程全面建成。青少年实践教育基地、市一中和夷陵中学新校区等重点项目加快推进。计划生育国策全面贯彻落实。城乡医疗卫生服务体系不断完善。人均预期寿命达到77.28岁。成为全省首批健康城市。

　　三峡工程全面建成，服务三峡工程安全运行成效明显。国防动员、民兵预备役建设和“双拥”工作不断加强，蝉联五届“全国双拥模范城”。物价、供销、粮食、统计、对台、外事、侨务、民族、宗教、社科、保密、信访、档案、方志、科普、妇女、儿童、援藏、援疆等各项工作都取得新成绩，气象、防震减灾、人防、老龄、残疾人等各项事业都取得新进步。

　　过去五年，是民主法治大进步的五年。自觉接受市人大及其常委会的法律监督、工作监督，主动接受市政协的民主监督，虚心听取各民主党派、工商联、无党派人士和广大市民的意见，按期办复人大代表议案和建议1390件、政协委员建议案和提案2385件。“六五”普法全面完成。全市90%以上单位成为法治创建合格单位，“民主法治示范村”创建率超过60%。办理法律援助案件1.1万件、法律援助事项10.2万件。平安宜昌建设成效明显，公众安全感和执法满意度连续三年居全省市州第一，荣获全国社会管理综合治理“长安杯”。安全生产形势总体稳定、持续好转。扎实开展党的群众路线教育实践活动和“三严三实”专题教育，推动作风建设制度化、常态化。党风廉政建设和反腐倡廉工作成效显著。行政监察和审计监督进一步加强。

　　刚刚过去的2015年，我们深入开展“新常态、新作为、新业绩”主题实践活动，统筹推进“稳、促、调、惠”各项工作，全市经济运行保持“稳中有升、稳中有进、稳中有转”的良好态势，延续“高于全国全省、领先中部沿江”的竞进势头，完成了市人大五届六次会议确定的主要预期目标。生产总值增长8.9%，工业增加值增长11.5%。地方公共财政预算收入增长24.9%，全社会固定资产投资增长20%，社会消费品零售总额增长13%，外贸出口增长19.3%，城乡居民人均可支配收入分别增长9%和9.7%。全年空气质量优良天数增加76天，优良率上升20.8个百分点。三峡经济枢纽新区等一批重大事项被纳入省“十三五”规划。中交建集团、广药集团等一批知名企业纷纷“加盟”宜昌。城市绿色发展、学习对接上海自贸区改革、棚户区改造、食品安全城市和农产品质量安全县创建等一批经验在全省全国推广。2015年目标全面完成，既实现了“十二五”规划圆满收官，又为“十三五”发展奠定了坚实基础。

　　各位代表，经过五年的改革发展，今天的宜昌，经济更加繁荣，社会更加和谐，环境更加优美，人民更加幸福，城市知名度和美誉度显著提高。这些成绩的取得，得益于省委省政府和市委的正确领导，得益于市人大、市政协的监督和支持，凝聚着全体代表、委员的智慧和力量，倾注了全市人民的心血和汗水。在此，我谨代表市人民政府，向全体人大代表、政协委员，向广大干部群众、离退休老同志，向各民主党派、工商联、无党派人士和社会团体，向驻宜部队官兵、中省驻宜单位，向所有关心、支持和参与宜昌建设的海内外朋友，表示衷心感谢并致以崇高敬意！

　　我们也清醒地看到，宜昌综合实力与省域副中心城市的定位还有一定差距；新旧动能尚未实现真正转换，资源环境约束加剧，经济提质增效扩量面临较大压力；制约经济社会发展的一些体制机制障碍依然存在；区域协调发展、城乡统筹发展的任务还很艰巨；生态建设和环境治理、民生改善和公共服务水平与群众期盼还有不小差距；政府依法行政水平和工作效能还有待进一步提高。对此，我们必须高度重视，采取更加有力的举措认真加以解决。

　　二、“十三五”时期发展目标与要求

　　今后五年，是全面建成小康社会的决胜期，也是现代化特大城市建设的关键期。国家重点实施长江经济带、“一带一路”发展战略，加快建设长江中游城市群，全面实施三峡后续工作规划，大力推进精准扶贫精准脱贫；省委省政府加快实施“一主两副多极”、“两圈两带一群”战略，支持申报建设湖北自贸区宜昌片区；三峡综合交通运输体系进入国家战略，三峡城市群、三峡经济枢纽新区正在争取纳入国家战略。这些机遇叠加，为宜昌创造了良好的发展环境。近年来我们通过改革创新，在体制机制、产业发展、平台建设等诸多方面积累的发展能量、创造的比较优势，为宜昌增添了新的发展动力。全市上下政通人和、心齐气顺、干事创业，形成了强大的发展气场。站在新的历史起点，我们对宜昌未来充满信心。

　　根据市委五届六次全会精神，“十三五”时期，我们将全面贯彻党的十八大和十八届三中、四中、五中全会精神，深入贯彻习近平总书记系列重要讲话精神，围绕“四个全面”战略布局宜昌实施，全面落实创新、协调、绿色、开放、共享发展理念，大力实施创新驱动、绿色繁荣、开放先导、依法治市、共建共享战略，坚持竞进提质、升级增效、以质为帅、量质兼取，加快建设湖北省域副中心城市、三峡城市群中心城市、长江中上游区域性中心城市，加快建设国家创新创业示范城市、国家生态文明建设先行示范城市、国家产业转型升级示范城市、长江流域区域开放合作示范城市和世界水电旅游名城，确保在全省率先全面建成小康社会、率先实现精准脱贫，综合实力进入中西部和长江沿线同等城市前列，进入全国城市50强，为建成“既大又强、特优特美”的现代化特大城市打下决定性基础。到2020年，力争生产总值、城乡居民人均可支配收入在2015年基础上翻一番；地方公共财政预算收入突破500亿元；固定资产投资突破7000亿元；规模工业总产值突破1万亿元；社会消费品零售总额突破2000亿元。

　　实现上述目标，我们将牢牢把握五点基本要求。

　　一是深入推进创新发展。深入实施高质量可持续发展核心战略，强化企业创新主体地位和主导作用，加快形成以创新为主要引领与支撑的经济体系和发展模式，把宜昌建设成为全域创新智谷和全国区域性创新创业之都。推动产业向中高端水平迈进，确保2020年现代服务业和高新技术产业增加值占生产总值比重分别达到35%和22%，形成精细化工、食品饮料、先进装备制造、新材料、节能环保与新能源、生物医药、新一代信息技术、现代物流、文化创意、旅游等千亿级产业集群，建成沿江万亿经济走廊，打造长江经济带重要增长极。

　　二是深入推进协调发展。坚持区域协同、城乡一体、两个文明并重、军民融合发展。完善和实施城乡全域规划，构建“一主六副、一带四廊”市域城镇空间布局，形成大、中、小城市和小城镇协调发展格局，加快推进以人为核心的新型城镇化。到2020年，全市常住人口和户籍人口城镇化率分别达到65%和45%。坚持“双轮驱动”，实现城区经济与县域经济齐头并进。促进城乡公共资源均衡配置、区域之间基础设施互联互通。建成全省首个“全国文明城市群”。打造全国重要的军民融合特色产业基地。

　　三是深入推进绿色发展。大力实施生态立市战略，倡导后现代、绿色生态、可持续的发展理念，坚持走生态优先、产城融合、城乡一体、人城共进、智慧个性、包容共享的绿色发展之路，加快建设“美丽宜昌”。加快推进主体功能区建设，不断完善绿色发展体制机制，深入推进绿色低碳循环发展，全面加强生态建设和环境治理，深入实施大气、水、土壤污染防治行动计划。按照“建设生态长江、涵养文化长江、繁荣经济长江”的思路，统筹推进宜昌沿江保护与发展。建成国家生态文明建设先行示范城市和全国绿色发展示范城市。

　　四是深入推进开放发展。深入实施开放先导战略，全面提升开放型经济水平，加快建设中西部地区开放型经济强市和长江流域区域开放合作示范城市。加快推进三峡综合交通运输体系、三峡城市群、湖北自贸区宜昌片区、三峡经济枢纽新区、“宜新欧”和“宜蒙欧”国际物流新通道等重大战略平台建设，构建陆水内外联动、东西双向开放的全面开放新格局，努力把宜昌打造成长江经济带对接“一带一路”的重要枢纽和战略支点。

　　五是深入推进共享发展。坚持“保基本、补短板、兜底线、促公平”原则，从解决人民群众最关心、最直接、最现实的利益问题入手，提供更充分、更均等的基本公共服务，促进社会公平正义，实现全市人民共同迈入全面小康社会。大力实施产业扶持、易地搬迁、生态补偿、教育脱贫、社保兜底等措施，坚决打赢脱贫攻坚战，确保“精准扶贫、不落一人”。加快建设宜业、宜居、宜旅、宜学、宜养之城，使全体人民在共建共享发展中有更多获得感。

　　三、2016年工作重点

　　今年是“十三五”规划实施的第一年，开好头，起好步，至关重要。全市经济社会发展主要预期目标是：生产总值增长10%，规模工业增加值增长10%，地方公共财政预算收入增长12%，固定资产投资增长18%，社会消费品零售总额增长13%，外贸出口增长12%，城乡居民收入增速与经济增长同步，居民消费价格涨幅控制在3%以内，城镇登记失业率控制在4.2%以内，全面完成省政府下达的节能减排和环境保护任务。

　　围绕实现上述目标，重点抓好以下七方面工作。

　　（一）突出改革创新，着力增强发展活力和动力

　　深化重点领域改革。全面落实中央、省关于供给侧结构性改革的各项部署，抓好去产能、去库存、去杠杆、降成本、补短板工作，促进新旧动能加快转换。着眼于简政放权，扩权强县、扩权强区，进一步精简市级审批事项，加强事中事后监管，推动政府职能转变。加强市县乡村政务服务体系标准化建设，大力推进并联审批，全面推行网上审批。清理规范行政审批中介服务事项，公开服务事项目录、机构目录和收费目录。深化商事制度改革，创新服务方式。进一步整合公共资源交易平台，将土地使用权、矿业权出让、医用耗材招投标纳入统一交易平台。深化投融资制度改革，积极争取负面清单管理试点。加快建设社会信用体系，健全跨部门联动响应机制和失信惩戒机制，大力创建国家信用建设示范城市。深化财政改革，创新投入机制，规范政府债务管理，强化财政支出约束，提高资金使用绩效。不断深化国有企业改革，进一步提高国有资本证券化水平，探索“非转经”和经营性国有资产集中统一监管，提高国有资本运营效率。抓紧完成不动产统一登记制度改革。全面完成农村土地确权登记颁证工作，建立健全农村综合产权交易体系。推进乡镇“一府三中心”改革。深化科技计划管理改革，建立健全由市场决定技术创新项目和经费分配的机制。继续实施县级公立医院改革。积极推进教育、医疗、天然气、电力等重点领域价格改革，加强价格监管。做好机关事业单位养老保险制度改革。推进综合行政执法体制改革试点。深化事业单位分类改革。

　　全面实施创新驱动。创新产学研合作方式，提升与高校、科研院所协同创新合作水平，确保完成科技成果转化扩散项目100项以上。强化企业创新主体地位，支持安琪生物技术研究院、宜昌磁电子研究院、兴发磷化工研究院和南玻硅材料技术中心创建国家级研发平台。放大国家技术转移中部中心宜昌分中心带动效应。新建一批科技咨询、中介服务、标准创新、检测检验、产权交易等公共服务平台。大力发展高新技术产业，确保新增国家高新技术企业50家以上，高新技术产业增加值突破630亿元、增长15%。深入推进大众创业、万众创新，大力实施创新创业动力支撑、众创空间发展倍增、“双创”主体培育引进、创新型产业引领、财政金融创新助推、创新创业服务提升等“六大工程”，加快打造创新创业之都。新增孵化器面积10万平方米以上，在孵企业总数达到500家以上。确保求索·众创中心建成投入使用。加快建设知识产权强市，确保发明专利申请量和授权量增长10%以上。全面实施质量强企战略、标准化战略和精品名牌战略，加快实施服务质量提升工程，推进卓越绩效管理，支持企业争创政府质量奖。力争新增中国驰名商标4件以上。

　　积极扩大有效投资。坚持把投资和项目作为“补短板”的主抓手，突出现代产业、基础设施、公共服务三大领域，进一步提高投资的精准性，确保投资总规模突破3500亿元。谋划和争取一批重大项目纳入国家、省计划笼子。抓好提请省委省政府支持的5个重大事项、13个重大项目、11项重大政策的争取、落实工作。深入开展“重大项目建设年”活动，加快推进惠科电子、兰台科技、东土互联网产业化、南玻电子级多晶硅、凌云航空产业、宜化光固化新材料、兴发中低品位磷矿综合利用、东阳光仿制类药物等100个省市级重点项目建设，确保一批项目早建成、早见效。进一步完善重大项目推进机制，下大力解决好融资、用地、征迁等实际困难。放大三峡产业引导股权投资基金、城市建设发展基金引导作用，积极争取省长江经济带产业基金、省重大基础设施建设项目资金的支持，引导和带动社会资本加大投入，努力形成市场化、可持续的投入机制。加快推进首批28个市级PPP项目落地，抓紧推出新一批PPP项目。设立“宜昌PPP民生事业发展基金”，引导社会资本集中投向教育、医疗、养老、公共交通、食品药品安全和环境保护等六大民生领域，分期建设一批重点项目，逐步解决城区入托、上学、就医、养老、停车等难题。支持市级四大控股集团开展市场化融资，力争全年融资400亿元以上。

　　做强人才保障支撑。统筹推进“宜昌英才工程”，完善高层次人才引进政策，健全人才引进机制，大力引进各类高层次人才和团队。完善人才服务机制，提升“专家服务中心”服务功能，确保市人才公寓示范小区年内交付使用。统筹推进“111”企业家培育工程、新生代企业家“菁英计划”、名师名医名家培育工程、“金蓝领”开发工程、现代农业科技人才开发工程、社会工作人才开发工程等系列人才工程，切实抓好各类人才队伍建设。加强市高技能人才公共实训基地建设，力争纳入国家级公共实训基地范畴，新增高技能人才6000人。推进专业技术人才职称改革，优化技能鉴定服务方式，营造各类人才脱颖而出、才尽其用的环境。

　　（二）突出产业转型，着力提升发展质量和效益

　　增强工业核心竞争力。深入推进工业强市战略，更加注重工业化与信息化、制造业与服务业、军民产业“三个融合”，推动工业经济向中高端迈进，加快建设国家产业转型升级示范城市。积极对接“中国制造2025”和“互联网＋”行动计划，抓紧制定“中国制造2025宜昌行动纲要”，推动宜昌制造业向智能化方向发展。深度对接国家、省战略性新兴产业规划、政策和重大科技专项，加快发展生物医药、新一代信息技术、新材料、节能环保与新能源等新兴产业。实施金宝乐器立式钢琴、恒安药业原料药等246个技改项目和示范工程，引导企业积极运用新技术、新工艺、新管理、新模式。深化与中船重工、兵装集团、航天科工等单位合作，着力培育民用航空航天、海洋工程、“气化长江”装备、磁电子等百亿级特色产业集群，创建国家级军民融合产业示范园区。力争精细化工产业产值突破2000亿元，食品饮料产业产值突破1500亿元，先进装备制造产业产值突破1200亿元，新材料产业产值突破1000亿元，节能环保与新能源产业产值突破600亿元，生物医药产业产值突破500亿元，新一代信息技术产业产值突破300亿元。确保规模工业总产值突破6000亿元，新增规模工业企业130家。加大“去产能”力度，对不符合国家能耗、环保、质量、安全等标准和长期亏损的产能过剩行业企业，实行关停并转或剥离重组；对连续亏损三年以上且不符合结构调整方向的企业，通过资产重组、产权转让、破产清算，实行“腾笼换鸟”、市场出清。做实“降成本”工作，进一步落实好结构性减税、电价调整、降低社会保险费、取消和暂停征收涉企收费等政策措施，降低市场主体制度性交易成本。

　　突破性发展现代服务业。围绕打造长江三峡国际旅游目的地、中国休闲度假特色地、鄂西乡村旅游首选地目标，加快推进平湖半岛、三峡游轮中心建设，积极创建宜昌三峡国家级旅游度假区。加速完善西陵峡风景区、清江观光度假区、五峰生态度假区、百里荒休闲度假区等服务功能。加快远安、兴山、龙泉、安福寺、晓峰、下牢溪、车溪等特色乡村旅游区建设，推进乡村旅游服务标准化。支持三峡大瀑布创建国家5A级旅游景区。力争西陵营盘山旅游综合体、当阳关公文化旅游城、远安嫘祖故里文化村等重点项目竣工营运。全力创建全国旅游标准化示范城市。确保全年接待游客5200万人次，旅游总收入达到600亿元以上。围绕构建“1+10”现代物流产业体系，加快推进三峡翻坝物流园、白洋物流园等建设，启动建设枝城综合物流园、宜昌东站物流中心等11个物流项目，力争现代物流业产值达到1200亿元。推动文化与旅游、科技、体育等融合发展，确保文化创意产业总产值突破400亿元、增加值突破200亿元。积极创建国家电子商务示范城市，加快发展农村电商、跨境电商，大力扶持和培育一批电商示范企业，推动本地产品全网销售和个性定制。大力发展研发设计、技术服务、软件外包等新兴服务业，加快推进省级服务业综合改革试点区建设。确保全年新增“小进限”企业100家以上。大力发展健康养老产业，力争江南康养城项目开工建设。支持清江流域发展康养产业。贯彻落实国家、省出台的一系列房地产“新政”，满足新市民住房需求，畅通安置房、公租房与商品房供需通道，加快化解房地产库存，促进房地产市场持续健康发展。认真落实“金融十条”和加快保险业发展意见，力争新引进金融机构5家，新增上市或“新三板”挂牌企业10家以上。支持枝江开展全省农村合作金融创新试点。依法严厉打击非法集资、金融欺诈等行为，有效防范化解金融风险。进一步规范市场秩序，改善消费环境，加快打造全国最佳放心消费城市。

　　加快发展现代特色农业。着力构建现代农业产业、农业安全生产、农业科技支撑、三次产业融合、现代农业经营“五大体系”，加快建设特色农业、智慧农业、绿色农业、魅力农业、活力农业。着力发展柑桔、茶叶、畜牧产业，壮大发展蔬菜、水产、食用油产业，加快发展花卉苗木、中药材、食用菌产业。重点推动柑桔优质高效栽培集成技术应用、非标果利用和“零废弃”精深加工，力争柑桔产业综合产值突破200亿元。重点实施“提升绿茶、振兴红茶、发展黑茶”战略，全面推广绿色防控，主攻中端茶，争取“宜昌红茶”品牌全面运作。重点发展草食畜牧业，力争出栏肉牛12万头、肉羊200万只以上。围绕打造“千亿产业、千亿企业、千亿大县”，力争全市农产品加工产值突破2100亿元，与农业总产值的比例达到3.2：1。全面推进农业与旅游、康养等产业深度融合，加快建设宜都国家柑桔农业公园、夷陵“三峡茶谷”等一批现代农业休闲旅游综合体。加快建设国家柑桔工程研究中心、湖北茶产业研究院等农业创新平台。启动三峡果蔬交易中心等物流配送项目建设，支持三峡物流园、三峡国际旅游茶城等专业市场提档升级。建设高标准农田55万亩，改造渠系500公里，不断提高农业设施化、机械化水平和防灾减灾能力。加强农产品质量安全监管，在全市食用农产品生产企业、合作社、基地加快建立农产品质量安全诚信体系。

　　（三）突出开放合作，着力扩大发展优势和空间

　　提升开放型经济水平。全面对接国家战略，深度融入“一带一路”、长江经济带和长江中游城市群等重大战略布局，加强与上海自贸区对接互动，促进投资、贸易便利化。落实投资贸易促进政策，拓宽利用外资领域和渠道，力争全年实际利用外资增长10%。扩大精细化工、生物医药、装备制造、农产品等出口优势，提高自主品牌和服务贸易出口比例，推动外贸向优质优价、优进优出转变。启动综合保税区申报工作，推进湖北自贸区宜昌片区申报，力争开通“宜新欧”货运班列。支持宜昌名企、名品参加国际国内会展。支持三峡机场改造升级，增开国际航线。积极引入“关检合作”通关模式，加强口岸信息化、智能化建设，持续优化大通关环境。加快“走出去”步伐，鼓励企业到境外设立营销网络、研发机构，支持企业参与国际并购，开辟国际市场。加强外事工作，深化与国际友城交流合作，提升宜昌城市对外影响力。推进中外合作在宜建设新型产业园区。

　　加大招商引资力度。充分发挥宜昌的区位优势、资源优势、产业优势、环境优势，紧紧围绕千亿产业集群和新兴产业，重点瞄准沿海发达地区和高新技术产业聚集地区，紧盯世界500强、中国500强等优强企业和具有较强竞争力的优质项目，实施精准对接，不断提高招商成功率、项目落地率。大力开展招商策划、招商包装、招商营销，主动对接科研机构、高等院校和国内外展会、投资洽谈会等载体，推进“四大平台”与县市区联动招商。坚持以诚心诚信、高效服务开展招商工作，重点抓好网络招商、以商招商、驻点招商。确保全年招商引资实际到位资金1450亿元，新开工亿元以上招商项目350个。

　　深化区域互利合作。探索建立三峡城市群协调会商机制，推进区域内各城市在产业发展、交通运输、生态环保、文化教育等各领域对接合作。加强与长江三角洲、珠江三角洲、武汉城市圈、成渝城市群等交流合作。深化与三峡集团、中国联通、武钢集团、中建集团、中交建集团、葛洲坝集团、省交投集团、鄂旅投集团等中省企业战略合作，力争在综合交通、现代物流、基础设施、载电体产业等方面取得一批新的成果。支持山区县、库区县到沿江地区发展飞地经济，加快建设飞地产业园区。积极服务三峡工程安全运行。依托对口支援三峡库区各种平台，加强与上海市等支援方合作，办好对口支援经贸洽谈会，确保全年对口支援到位资金100亿元。加大援藏援疆工作力度，鼓励宜昌企业参与西藏、新疆建设发展。

　　（四）突出城乡统筹，着力提升城市功能和品质

　　实施新区建设攻坚行动。认真落实中央、省城市工作会议精神，贯彻“理念为先、以业兴城、规划统领、文化彰显”要求，继续在基础设施、大型公建、安置房、生态园林等领域，实施一批关键性、支撑性、引导性的重大项目，确保完成投资400亿元以上，为“五年成规模”打下决定性基础。加快实施至喜长江大桥扫尾工程，积极推进快速路网建设，确保上半年内环线闭合通车。确保宜昌BRT全线贯通运行。开工建设伍家岗长江大桥、西陵二路上段、峡州大道中段等项目，力争年底外环线基本成形。续建新建道路150公里，确保建成通车60公里。统筹推进教育、医疗、商业等配套设施建设，力争市档案馆新馆、科技馆、大剧院、音乐厅等项目年内开工。确保市博物馆新馆、奥体中心体育馆、市一中新校区、夷陵中学新校区、市委党校新校区、青少年实践教育基地、老年大学建成并投入使用。推进中建？宜昌之星、江南生态新城、环城南路历史文化风貌街区、宜昌国际广场等大型城市综合体加快建设。加快卷桥河湿地公园、求雨台公园等项目建设，确保磨基山公园、柏临河公园、城东公园、滨江公园升级改造等项目建成开放。确保年内形成城东片区、东站片区、唐家湾片区、奥体中心片区、卷桥河片区等成熟片区。

　　提升城市建设管理品质。实施“智慧宜昌”建设三年行动计划，积极开展智慧城市、信息惠民、宽带中国等试点示范，重点抓好光纤入户、4G网络全覆盖、城区公共场所免费WIFI全覆盖工程，以及三峡云计算中心、大数据中心等信息基础设施建设，加快推进智慧健康医疗、智慧食药监管、智慧环保等重大应用项目建设。力争宜昌“市民卡”年内实现主城区全覆盖。抓好城市修补，重点推进五一广场、中南路、九码头、西陵区沿江、夷陵广场、西陵二路沿线、红光港机、平湖和葛洲坝等九大片区棚户区改造。深入推进城市整理工程，对条件适宜的建筑楼顶推进“平改坡”，开展多层既有住宅加装电梯试点，确保全年完成建筑综合整治项目250个，逐步实现建成区主干道两侧既有建筑综合整治全覆盖。高水平发展城市公共雕塑艺术。抓好沙河片区、卷桥河片区“海绵城市”建设试点。基本建成城区地下管线信息管理系统。加快推进将军路、江南二路等地下综合管廊建设。深入推进城市执法体制改革，以法治化、精细化、智慧化、人本化为方向，进一步改进城市管理工作。开展“深度保洁、以克论净”环卫试点。提高道路清扫机械化作业水平。加强停车场建设及道路停车规范管理。开展摩托车、电动车、非机动车“三车”常态化整治，规范城区道路交通秩序。优化城区公交线网布局，抓好BRT管理运营，改善公交换乘条件，让市民出行更方便。坚决治理污染、拥堵、内涝等“城市病”。加强和规范住宅小区物业管理。

　　统筹发展城区经济和县域经济。加快宜昌高新区电子信息产业园、生物产业园、白洋工业园等“一区七园”基础设施建设，确保新引进项目40个以上，完成投资480亿元，加快打造全市经济发展的核心引擎。支持西陵区加快建设金融服务、创新创业、科教文化中心。支持伍家岗区加快发展总部楼宇经济、金融服务业、高新技术产业、现代商贸服务业。支持点军区加快发展新一代信息技术、文化旅游、康养产业。支持猇亭区加快建设猇亭工业园、深圳工业园和临空工业园。支持夷陵区加快建设全省城乡统筹发展示范区。推动产业、项目、基础设施市县对接，促进城乡要素平等交换、合理配置。支持宜都、枝江、当阳、远安争先进位，加快发展。支持长阳、秭归、五峰、兴山脱贫攻坚，奋力赶超。进一步完善户籍制度改革，促进有能力在城镇稳定就业和生活的农业转移人口进城落户。支持宜都开展国家新型城镇化试点。加快11个新型城镇化试点镇、9条统筹城乡发展示范带建设。加快农村电网改造升级，实施秭归220千伏输变电等15个电网重点工程。

　　进一步完善城乡交通网络。加快三峡综合交通运输体系、内畅外联网络体系建设，完成交通投资115亿元。加快推进宜昌至来凤高速公路前期工作，启动神农架至五峰高速公路、枝江至石门高速公路、沪蓉高速公路宜昌横向连接线方案研究。开工建设宜张高速五峰至湘鄂界段、江北翻坝高速公路、点军联棚至长阳龙舟坪一级公路，加快建设宜昌江北快速通道、宜昌至黄花等5条一级公路，建成五峰王家冲至小河等5条一级公路，力争全年建成一级公路50公里。推进城乡道路客运班线公交化改造。力争新开通城际公交线路2条。开工建设白洋长江大桥，加快建设香溪长江大桥。续建云池港二期、茅坪港二期等8个项目，启动白洋港二期、枝城铁水联运码头等6个项目建设，着力完善三峡枢纽港集疏运功能。积极推进宜昌至襄阳、宜昌东至郑万铁路连接线、宜昌至常德3个高铁项目前期工作。力争开工建设远安至当阳地方铁路、三峡铁水联运铁路。加快推进轨道交通2号线项目前期工作，开工建设市域铁路东站至小溪塔改造项目。

　　（五）突出绿色生态，着力加强环境保护和治理

　　狠抓重点领域污染防治。严禁新上高污染高耗能项目，严格控制主要污染物排放。推进工业废气治理，强化机动车尾气、建筑扬尘等污染防治，落实秸秆综合利用政策和禁烧措施，完成黄标车、老旧车淘汰任务，确保全年空气质量优良天数超过70%。实施全市水污染防治行动计划，确保国家和省考核断面水质达标。全面实行“河长制”。加快推进长江宜昌段干支流综合治理。抓好三峡库区截污清漂工作。加大黄柏河流域、清江流域综合防治力度。完成沮漳河等6条中小河流年度治理任务。严格涉磷企业监管。严格集中式饮用水源地保护。依法取缔水库投肥养殖和江河湖库围栏围网养殖。加快实施沙河、柏临河、紫阳河等流域综合整治工程。加强城市污水收集管网建设和沿江排污口治理，2017年基本消除城市建成区黑臭水体。建成卷桥河污水处理厂一期工程。严厉打击长江非法采砂。治理水土流失面积300平方公里，加强地质灾害防治。推进宜都、长阳省级水生态文明建设试点。抓好重金属污染物等专项整治和土壤修复试点。全面开展生活垃圾分类工作。加大农业面源污染防治力度，统筹推进农村改水改厕、畜禽养殖、垃圾处理。加强环境监管能力和环境应急能力建设，坚持依法治污、铁腕治污，努力提高人民群众对环境质量的满意度。

　　深入推进绿色宜昌建设。巩固提升国家森林城市创建和全省城市绿色发展现场会成果，努力争创国家生态园林城市、国家生态文明建设示范市。加快推进“绿满荆楚”和绿化美化行动，突出抓好干道沿线、长江岸线、旅游观光线等重点区域绿化美化工作。全年植树造林15万亩，城区定植绿化大苗2.5万株，新建生态公益林11.1万亩，完成天保工程二期森林管护任务1166万亩。完善城市绿地系统和湿地保护系统，实施“三小”绿地、公园绿地、城市湿地、道路景观等改造修复和提升工程，建设城市绿道500公里。加快推进点军片区、宜昌东站片区、西陵唐家湾片区等绿色生态示范区建设，重点城建项目、宜昌新区新建建筑全面执行绿色建筑标准，全年新增绿色建筑100万平方米。加快推进农村清洁能源、生态家园、清洁田园、美丽乡村建设。

　　全面节约和高效利用资源。实行能源和水资源消耗、建设用地等总量和强度双控行动。加快风能、太阳能、生物质能利用项目建设，推进页岩气开发前期工作，推广使用清洁能源，积极创建国家新能源示范城市。大力实施一批循环经济、清洁生产和节能降耗示范工程。继续推进猇亭园区循环化改造试点，支持点军、长阳建设国家可持续发展示范区、试验区，支持枝江建设国家循环经济示范市。严守耕地保护红线，强化土地批后监管，加大闲置和低效利用土地清理处置力度，推广梯级土地开发模式，争创全国国土资源节约集约模范市。加强生态文明宣传教育，不断增强全民生态意识。

　　（六）突出共建共享，着力改善人民生活和福祉

　　全力实施精准扶贫。全面贯彻落实全国全省扶贫开发会议重要精神，将脱贫攻坚作为第一民生工程，按照“六个精准”和“五个一批”要求，推进专项扶贫、精准扶持到村到户到人。加大贫困地区道路、水利、电力、通信等基础设施建设力度。按照不低于地方财政收入增量的15%增列专项扶贫预算，建立扶贫投入县级整合机制。全面实施88个贫困村整村脱贫规划，实施第二批特困村脱贫攻坚行动。创新扶贫方式，积极发展金融扶贫、电商扶贫、旅游扶贫和资本扶贫。扎实推进易地搬迁扶贫工作。全面推行精准扶贫网格化管理。启动点军城乡统筹精准扶贫示范区建设。建立年度脱贫攻坚报告和督察制度，推动各项脱贫攻坚措施落地生根，确保年内10万人稳定脱贫。

　　切实加强民生保障。全面落实各项就业扶持政策。新增小额担保贷款2.7亿元，扶持创业4000人。加快全国公共就业管理服务标准化试点建设。实施高校毕业生就业促进和创业引领计划，加强困难群体就业帮扶，促进农村富余劳动力转移就业，鼓励农民工返乡创业，多渠道安置残疾人就业。确保城镇新增就业7.7万人，失业人员再就业2.5万人。实施全民参保登记计划，稳步扩大社会保险覆盖面。稳步推进城镇居民医保和新农合制度整合，建立统一的城乡居民医保制度。深化按病种、总额预付等医保付费方式改革，提高基金使用效率。稳步提高社会救助、优抚安置、社会福利等社会保障水平。加快食品药品监管信息化项目建设，实施“放心早餐工程”，抓好重点领域和重点环节食品安全专项治理，加快创建以国家食品安全城市为引领的“食品安全城市群”。建设一批放心粮油示范店。实施棚户区改造38226户，筹集公租房2618套。改造农村危房1.2万户。实施农村10万人饮水安全巩固提升工程。扎实推进三峡后续工作和水库移民后期扶持工作，促进移民同步小康。

　　全面发展社会事业。大力培育社会主义核心价值观，巩固全国文明城市创建成果，深入推进“全国文明城市群”建设，广泛开展“十星”系列创建活动。加快推进国家公共文化服务体系示范区建设，提升“诗歌之城”、“读书之城”、“钢琴之城”城市文化品牌。持续推进文化惠民工程，推出一批文艺精品。启动滨江文化城建设。积极支持三峡大坝和屈原故里打包申报世界文化遗产。办好屈原故里端午文化节、第六届宜昌长江钢琴音乐节、首届中国宜昌国际马拉松赛等节赛活动。加快推进广播电视“村村响”工程。全面实施第二期学前教育、特殊教育、教育信息化等“三年行动计划”，启动区域教育现代化创建工程，深入实施城乡教师支持计划。全面实施现代职业教育质量提升工程，推动职业院校产教融合、校企合作。加快推进高等教育发展，谋划建设科教城。支持三峡大学建设一流大学、一流学科。积极申办三峡航空学院，启动市特校新校区建设。大力开展各类社会教育，加快构建终身教育体系。完善城乡医疗卫生服务体系，确保市中心医院江南院区基本建成，加快三峡大学仁和医院东站院区、市妇幼医院、市中医院综合大楼等项目建设，加快打造区域性医疗卫生中心。支持乡村医生定向培训基地建设。全面推广分级诊疗、双向转诊“互联网+”模式。继续推进“改善医疗服务行动”。完善健康管理服务体系，积极创建“全国健康城市”。实施全面两孩政策。做好国家卫生城市复评工作。加快市妇女儿童活动中心建设。推进民族团结进步工作。依法管理宗教事务。推动乡村志编纂工作。继续抓好统计、供销、科普、社科、慈善、侨务、港澳、对台、保密、消防、气象、人防等各项工作。加强国防动员和后备力量建设，推动“双拥”工作再上新台阶。

　　（七）突出职能转变，着力提升治理能力和水平

　　深化创新社会治理。健全统筹城乡的网格化管理体系、广泛多层的社会化服务体系、协商共治的规范化自治体系和联通共享的信息化支撑体系，着力打造社会建设国家典范。加快建设农村网格“四务通”，提升城乡网格服务精准化、精细化水平。完善立体化社会治安防控体系，严厉打击各类违法犯罪，加强寄递物流安全管理，加快建设“平安宜昌”。抓好全国公共安全视频监控建设联网应用工程和全国吸毒人员网格化服务管理试点。加强法制宣传教育，加快建设法治宜昌。全面启动“七五”普法，大力开展法治县乡、法治单位等创建活动。完善人民调解、行政调解、司法调解工作体系。做好法律援助、司法救助等工作。加强和改进信访工作。充分发挥工会、共青团、妇联、工商联、残联等群团组织在服务经济发展、创新社会治理中的重要作用。加强应急管理和防灾减灾体系建设。加强全民安全意识教育，健全公共安全体系，落实安全生产主体责任，坚决遏制重特大安全事故发生。

　　全面推进依法行政。严格落实《法治政府建设实施纲要》，加快建设法治政府。积极协同人大做好立法工作，认真做好政府规章制定工作。完善干部学法用法制度，提高法治素养与依法办事能力。积极参加全市干部公共管理课程培训。强化依法、科学、民主决策，健全公众参与、专家论证、风险评估、合法性审查和集体讨论决定的重大行政决策机制，认真落实决策责任追究制度。严格行政执法，提高执法水平和效率。加强行政复议和应诉工作。自觉接受人大及其常委会法律监督和工作监督，主动接受政协民主监督，认真办好议案提案，广泛听取民主党派、工商联、无党派人士和人民团体意见。加强电子政务建设，实现政务行为痕迹化监管。抓好政府信息公开，加强政策宣传解读，保障公民的知情权、参与权、表达权、监督权。

　　加强政府廉政建设。严格执行关于改进工作作风、密切联系群众各项规定，巩固提升群众路线教育实践活动和“三严三实”专题教育成果。始终把纪律和规矩挺在前面，认真贯彻执行《中国共产党廉洁自律准则》和《中国共产党纪律处分条例》，严格落实党风廉政建设主体责任和“一岗双责”。加快编制政府部门“权责清单”，明确监管事项和监管责任，强化权力运行制约和监督。强化对财政资金分配使用、政府投资、公共资源转让、公共工程建设等权力集中部门和岗位的审计监督。对公共资金、国有资产、国有资源和领导干部履行经济责任情况实行审计全覆盖。强化政务督查和行政监察。支持纪检监察机关履行监督责任，严肃查处各类违法违纪案件。

　　各位代表！大城已呈腾飞势，风劲恰是扬帆时。让我们在省委省政府和市委的坚强领导下，与全市人民一道，以更加饱满的热情、更加昂扬的斗志、更加务实的作风，践行新理念、决战“十三五”，为实现“两个率先、两个进位”的宏伟目标、加快建设“既大又强、特优特美”的现代化特大城市而努力奋斗！
